# What the VAP: The Expanded VAP Family of Proteins Interacting With FFAT and FFAT-Related Motifs for Interorganellar Contact

**DOI:** 10.1177/25152564211012246

**Published:** 2021-05-09

**Authors:** Jacques Neefjes, Birol Cabukusta

**Affiliations:** Cell and Chemical Biology, Oncode Institute, Leiden University Medical Center, Leiden, the Netherlands

**Keywords:** VAP, MOSPD, FFAT, FFNT, membrane contact sites, endoplasmic reticulum

## Abstract

Membrane contact sites are formed by tether proteins that have the ability to bring two organellar membranes together. VAP proteins are a family of endoplasmic reticulum (ER)-resident tether proteins specialized in interacting with FFAT (two phenylalanines in an acidic tract) peptide motifs in other proteins. If the FFAT-motif-containing proteins reside on other organelles, VAP proteins form contact sites between these organelles and the ER. The role of VAPA and VAPB, the two founding members of the VAP family in recruiting proteins to the ER and forming membrane contact sites is well appreciated as numerous interaction partners of VAPA and VAPB at different intracellular contact sites have been characterized. Recently, three new proteins -MOSPD1, MOSPD2 and MOSPD3-have been added to the VAP family. While MOSPD2 has a motif preference similar to VAPA and VAPB, MOSPD1 and MOSPD3 prefer to interact with proteins containing FFNT (two phenylalanines in a neutral tract) motifs. In this review, we discuss the recent advances in motif binding by VAP proteins along with the other biological processes VAP proteins are involved in.

## Introduction

Eukaryotic life is defined by the presence of membranelimited organelles that are specialized in a multitude of biochemical processes. These organelles need to communicate with each other at membrane contact sites (MCS) to function properly (Wu et al., 2006; Rowland et al., 2014; Cabukusta and Neefjes, 2018; Spits et al., 2021). MCS are intracellular regions where two organelles are closely juxtaposed to form an intracellular synapse to facilitate interorganellar communication and metabolic exchange (Eisenberg-Bord et al., 2016; Gatta and Levine, 2017; Scorrano et al., 2019). While MCS are microdomains with defined proteomes and lipidomes, their formation is mediated by tether proteins that interact with specific proteins or lipids on opposing membranes (Vance, 1990; Garofalo et al., 2016; Scorrano et al., 2019).

The endoplasmic reticulum (ER) spans the entire cytoplasm and contacts virtually every membranebound organelle, the plasma membrane, and even membraneless organelles (Ma and Mayr, 2018; Wu et al., 2018; Scorrano et al., 2019; Lee et al., 2020). A significant portion of ER contact sites are formed by the ER-resident tether proteins VAPA and VAPB that interact with partner proteins located on other organelles (Wyles et al., 2002; Hanada et al., 2003; Wyles and Ridgway, 2004; Amarilio et al., 2005; Lehto et al., 2005; Loewen and Levine, 2005; Rocha et al., 2009; Mesmin et al., 2013). The role of VAPA and VAPB in forming contact sites is well appreciated and new interaction partners of VAPA and VAPB are unveiled each year (Lindhout et al., 2019; Nthiga et al., 2020; Zhao et al., 2020; Inukai et al., 2021). Recent work from us and others unravelled three new human homologs of VAPA and VAPB, namely MOSPD1, MOSPD2, and MOSPD3, that also form MCS (Di Mattia et al., 2018; Cabukusta et al., 2020). Along with an expanding VAP family, the number of motifs that can be recognized on target proteins also multiplied. In this review, we address the most recent advances in motif binding, protein recruitment, and contact site formation by VAPA, VAPB, MOSPD1, MOSPD2, and MOSPD3, hereafter collectively referred to as VAP proteins. This is followed by a discussion of various biological processes, including genetic and infectious diseases VAP proteins are involved in.

## Multiple VAPs and FFAT Motifs

**Figure 1:**
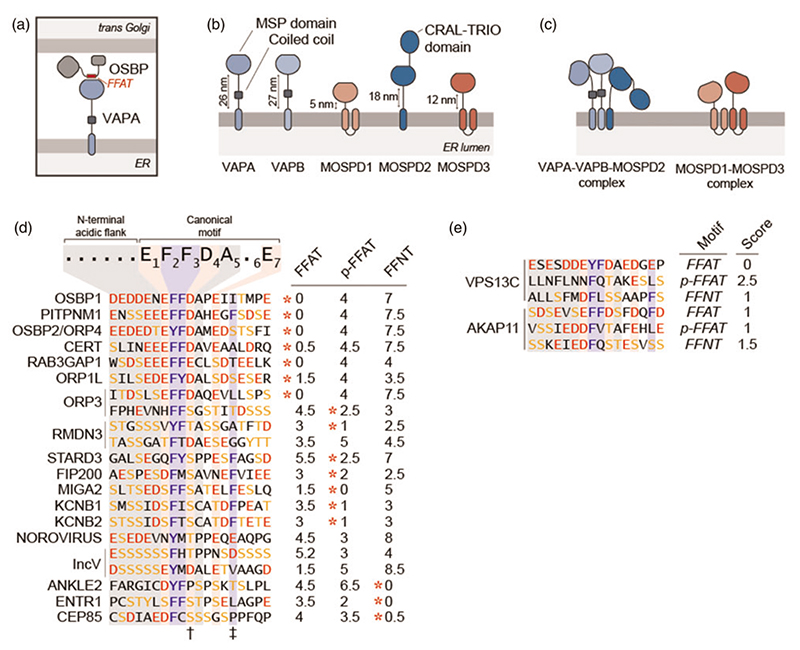
Human proteome contains multiple VAP proteins and FFAT motifs. (a) Schematic representation of VAP proteins forming MCS. ER-localized VAPA interacts with FFAT motif of Golgi-bound OSBP to create MCS between two organelles. (b) Human genome encodes five MSP-domain-containing VAP proteins that localize in the ER. The lengths of the linker regions between transmembrane helices and MSP domains are different in VAP proteins. Note that only VAPA and VAPB contain predicted coiled coil regions. (c) VAP proteins form two separate protein complexes in the ER as VAPA-VAPB-MOSPD2 and MOSPD1-MOSPD3 complexes. (d) The canonical FFAT motif contains the E-F-F-D-A-X-E consensus sequence preceded by acidic residues. Shortlist of proteins with reported FFAT and FFAT-related motifs. The panel on the right depicts the FFAT, phospho-FFAT (p-FFAT) and FFNT scores of each sequence. The score values represent the divergence of the sequences from the defined canonical motifs, e.g. OSBP, contains the canonical FFAT, has the score of 0. The motif the sequence is reported to belong is shown by a red asterisk. Note that RMDN3 and IncV contain tandem FFAT/FFAT-related motifs. The position 4 of the motif requires phosphorylation in phospho-FFAT (shown with a dagger^†^). The phenylalanine at the position 9 is accommodated in the secondary hydrophobic pocket of MOSPD2-MSP (shown with a double dagger‡). (e) Two examples, VPS13C and AKAP11, of proteins predicted to contain all three FFAT-related motifs.

The ER proteins VAPA and VAPB are specialized in recruiting other proteins to the ER and they often form MCS between the ER and other organelles (Figure 1a). VAPA and VAPB are highly similar in amino acid sequence and topology, both are singlespan membrane proteins with a coiled coil region and an MSP domain (Nishimura et al., 1999) (Figure 1b). The MSP domains of VAPA and VAPB interact with FFAT motifs in target proteins to bring them to the ER (Loewen and Levine, 2005). This then allows the formation of contact sites when the recruited proteins are associated with another organelle.

FFAT, two phenylalanines (FF) in an Acidic Tract, motifs are short linear peptide motifs with an E_1_-**F_2_-F_3_-** D_4_-A_5_-X_6_-E_7_ consensus core sequence preceded by an adjacent acidic flanking region (Loewen et al., 2003) (Figure 1d). While the canonical FFAT motif, EFFDAXE, is found in human and yeast proteomes, most motifs shown to interact with VAPA or VAPB deviate from the canonical motif in their core and/or acidic flanking regions (Slee and Levine, 2019). In fact, FFAT motifs can show variation in each of the seven core elements (Mikitova and Levine, 2012). Consequently, it remains essential to this date that all predicted motifs are tested experimentally.

As FFAT motifs can show countless variations, some appear more frequently than others. One of the best examples is the substitution of the acidic residues (aspartic acid or glutamic acid) with residues that can be phosphorylated to gain a negative charge, often a serine or a threonine. Indeed recently, Di Mattia *et al*. showed that the FFAT motifs of STARD3 (MLN64), MIGA2, FIP200 (RB1CC1), PTPIP51 (RMDN3), KCNB1 and KCNB2 contain a serine or a threonine at the 4th position of the motif (Di Mattia et al., 2020) (Figure 1d). The phosphorylation of this residue is required to interact with VAPA and VAPB and therefore indispensable for creating MCS. These FFAT-related motifs that require phosphorylation to interact with VAPA and VAPB are named phospho-FFAT motifs (Di Mattia et al., 2020). It is possible that some proteins contain both a conventional FFAT and a phospho-FFAT motif. For instance, OSBL3 uses both of its FFAT and phospho-FFAT motifs to create contact with the plasma membrane (Weber-Boyvat et al., 2015). Overall, the characterization of phospho-FFAT motifs implies that the formation of VAP-mediated contact sites can be controlled by kinases and phosphatases and ultimately by signal transduction.

**Figure 2:**
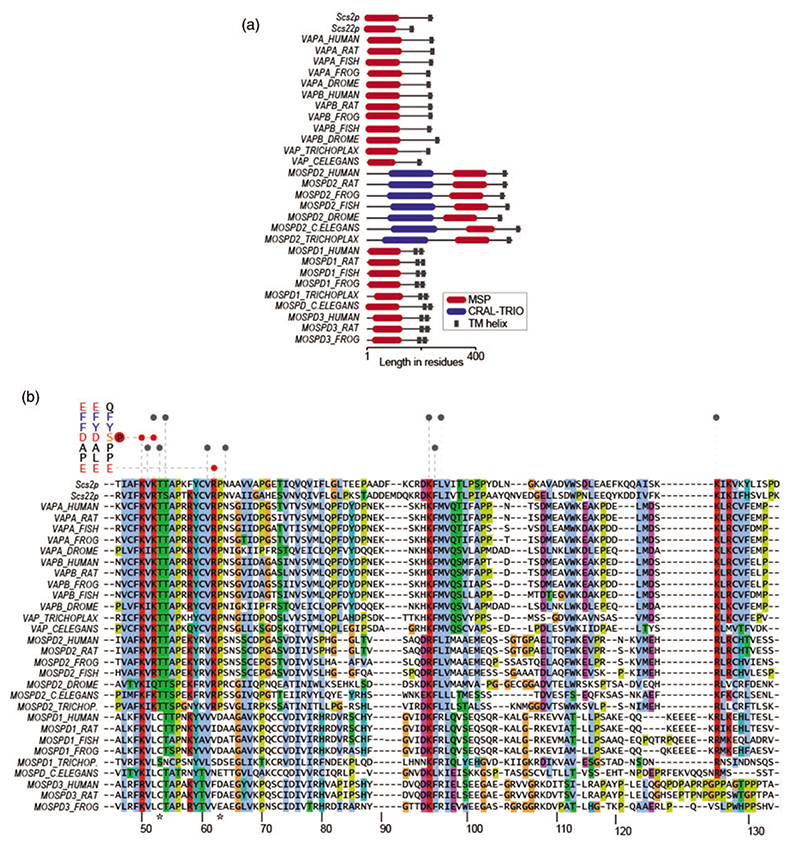
VAP proteins demonstrate varying levels of conservation. (a) Domain architecture of VAP homologs in various species from the evolutionary tree. (b) Alignment of VAP-MSP domains from various species, including Scs2p and Scs22p from S. *cerevisiae*. The interaction map of VAPA/MOSPD2 with FFAT and phospho-FFAT is depicted at the top. Red are electrostatic and grey are hydrophobic interactions. Mutations in T46 and P56 in VAPB causes familial ALS (shown with asterisks). Residue numbers for human VAPA are shown at the bottom.

The observations that MCS between the ER and other organelles persist even in the absence of VAPA and VAPB implied the presence of other scaffolds at these sites (Dong et al., 2016; Eden et al., 2016). Supporting this notion, MOSPD2 was identified as a third FFAT-motif-binding protein (Di Mattia et al.,2018). MOSPD2 also contains an MSP domain and the residues critical for FFAT binding are conserved among VAPA, VAPB and MOSPD2 (Figure 2b). Consequently, MOSPD2 also interacts with FFAT and phospho-FFAT motifs (Di Mattia et al., 2018, 2020). Despite this, VAPA, VAPB and MOSPD2 are not redundant tethers. As the depletion of both VAPA and VAPB reduces the extent of ER-endosome contact sites, MOSPD2 depletion has an even stronger effect on these sites (Di Mattia et al., 2018). This suggested that VAPA, VAPB and MOSPD2 have distinct functions at interorganellar contact sites.

The discovery of the third FFAT-motif-binding protein raised the question whether more motif-binding MSP domains are present in the human proteome. This led to the characterization of MOSPD1 and MOSPD3 with functional MSP domains (Figure 1b) (Cabukusta et al., 2020). The MSP domains of MOSPD1 and MOSPD3 are diverged from the MSP domains of VAPA, VAPB and MOSPD2, which suggested that these domains might bind motifs different from FFAT (Figure 2b). The motifs MOSPD1 and MOSPD3 interact with could be predicted by the available FFAT motif search algorithm (Slee and Levine,2019). Further analyses showed that the FFAT-related motifs favoured by MOSPD1 and MOSPD3 lack the acidic characteristics of FFAT but rather contain neutral amino acids and are thus called FFNT (two phenylalanines (FF) in a Neutral Tract) motifs (Figure 1d) (Cabukusta et al., 2020).

Since both FFAT and FFNT motifs can show countless variations, some sequences can be defined both as a FFAT and an FFNT motif. Theoretically, such sequences could be recognized by all five VAP proteins. Moreover, as it is possible for some proteins to carry a FFAT and a phospho-FFAT (such as OSBL3), it could be that some proteins contain both (phospho-)FFAT and FFNT motifs and interact with all VAP proteins (Figure 1e).

In addition to their ability to recruit proteins, each VAP protein can form homomeric and heteromeric protein complexes (Nishimura et al., 1999; Kim et al., 2010; Cabukusta et al., 2020). Moreover, their ability to form heteromeric complexes reflects their motif preferences (Figure 1c). In other words, VAPA, VAPB and MOSPD2, which prefer (phospho-)FFAT, interact with each other (Figure 1c). On the other hand, FFNT-favouring MOSPD1 and MOSPD3 form a separate complex (Cabukusta et al., 2020). Therefore, two segregated tethering complexes in the ER interact with different protein motifs and thus can form different intercompartment interactions (Figure 1c).

It is worthwhile to mention that different VAP proteins and their corresponding motifs display different levels of conservation throughout evolution. The yeast genome encodes two VAPA/VAPB homologs, Scs2p and Scs22p, as well as numerous proteins containing FFAT motifs. VAPA/VAPB homologs are also present in plants interacting with FFAT-related motifs (Saravanan et al., 2009). Meanwhile, MOSPD1, MOSPD2 and MOSPD3 emerge later in evolution as they are not found in yeast nor plants (Figure 2a). MOSPD1 and MOSPD2 appear in metazoans and can be found even in the lowest metazoan Trichoplax. MOSPD3 emerges later, only in chordates. In the meantime, all VAP proteins are broadly expressed in human tissues (Cabukusta et al., 2020). These might simply imply that complex life requires a complex organization of its interorganellar interactions.

## Selectivity and Mechanism of Motif Binding

VAP proteins form two segregated protein complexes in the ER. These consist of VAPA-VAPB-MOSPD2 and MOSPD1-MOSPD3 complexes specialized in interacting with (phospho-)FFAT and FFNT motifs, respectively. Beyond the selectivity for FFAT and FFNT motifs, an additional layer of selectivity emerges within these VAP complexes. This selectivity has been suggested earlier by Baron *et al*. showing that the FFAT-motif-containing proteins WDR44 and RAB3GAP1 prefer VAPB over VAPA (Baron et al., 2014). Also, we and others demonstrated that VAPA and VAPB have higher affinities towards the (phospho-)FFAT motifs of OSBP, CERT, PTPIP51 (RMDN3), KCNB1 and KCNB2 than MOSPD2 (Cabukusta et al., 2020; Di Mattia et al., 2020). While it remains unclear how the selectivity of VAP proteins is achieved, crystal and NMR structures of VAPs in complex with motifs visualize the molecular basis of these interactions (Figure 2b) (Kaiser et al., 2005; Furuita et al., 2010; Di Mattia et al., 2020).

The interaction between the MSP domain of VAPA and a FFAT motif begins with the acidic elements of the motif making non-specific electrostatic interactions with the positively charged surface of the MSP domain (Furuita et al., 2010). This interaction is later stabilized by more specific interactions: the phenylalanine at position 2 (F_2_) of the FFAT motif binds into a hydrophobic pocket of MSP created by the aliphatic parts of the sidechains from K52, T54, K94, M96, and K125 of VAPA, and A_5_ of the FFAT motif sits in a hydrophobic pocket created by the sidechains of V51, T53, V61, N64 and F95 (Figure 2b) (Kaiser et al., 2005; Furuita et al., 2010; Di Mattia et al., 2020). In the case of phospho-FFAT, phospho-S_4_ makes electrostatic interactions with K50 and K52 of VAPA, as the side chain of phospho-S4 is longer than that of D_4_ of ORP1L-FFAT motif to reach those residues. Accordingly, the mutation of the K50 residue of VAPA is sufficient to block its interaction with phospho-FFAT without affecting interactions with the ORP1L-FFAT motif (Di Mattia et al., 2020).

The interactions between the MSP domain of MOSPD2 with the ORP1L-FFAT and STARD3-phospho-FFAT motifs are homologous to the interactions with the VAPA-MSP domain (Di Mattia et al., 2020). Nevertheless, small differences between the MSP domains of VAPA and MOSPD2 still exist. Firstly, the mutation of MOSPD2 K363, corresponding to K50 in VAPA, does not block the interactions with either FFAT or phospho-FFAT, but affects the interactions only mildly. In addition, the MOSPD2 MSP domain contains a secondary hydrophobic pocket formed by N378, P420, L423, and T424 in which F_9_, two residues downstream of the core motif, of STARD3-phospho-FFAT can be accommodated. It is, therefore, possible that MOSPD2 specializes in interacting with motifs containing a phenylalanine residue at position 9 in the FFAT motif. However, this appears more complicated as the phospho-FFAT motifs with this feature, KCNB1 and KCNB2, do not interact with MOSPD2 (Figure 1d) (Di Mattia et al., 2020). This further points out that there is yet no absolute rule to determine VAP motif selectivity and highlights the intricate nature of motif selectivity among VAP proteins.

When the residues directly involved in interacting with the FFAT core motifs are compared, MOSPD1 and MOSPD3 MSP domains diverge from the VAPA, VAPB and MOSPD2 MSP domains. The majority of the residues forming the hydrophobic pocket that accommodate F_2_ and A_5_ are conserved in MOSPD1 and MOSPD3: V51, T54, V61, N64, K94 and F95, and K125 in MOSPD1 (Figure 2b). The residues forming electrostatic bridges with the acidic elements of the FFAT motif are somewhat less conserved, such as K52 and R62. This corresponds with the observation that FFNT motifs as preferred by MOSPD1 and MOSPD3 have fewer acidic elements. Overall, more in-depth structural studies are required to resolve the molecular details that determine the motif selectivity.

## Differences Besides Motif Binding

Despite sharing the same subcellular localization with a similar membrane topology and interacting with short linear motifs, the five VAP proteins also show differences. Notably, MOSPD2 is the only member with an additional domain, a CRAL-TRIO domain (Figures 1b and 2a). Characteristically, CRAL-TRIO domains contain a hydrophobic pocket allowing interactions with lipids and other small hydrophobic molecules. The yeast CRAL-TRIO-containing Sec14p is a phosphatidylinositol (PI)/phosphatidylcholine (PC) transfer protein that is essential for protein transport from the Golgi complex to the plasma membrane (Bankaitis et al., 1989, 1990). Other CRAL-TRIO domains of yeast were also reported to interact with phospholipids (Schaaf et al., 2008; Yang et al., 2013). Mammalian CRAL-TRIO domains are reported to bind a variety of lipids. Neurofibromin CRAL-TRIO interacts with PC, PI, phosphatidylglycerol (PG), phosphatidylserine (PS), and phosphatidylethanolamine (PE) and Clavesin-1 binds to PI-3,5-bisphosphate (PI-3,5-P_2_) (Welti et al., 2007; Katoh et al., 2009). In addition to phospholipids, mammalian CRAL-TRIO domains can interact with small hydrophobic molecules. The CRAL-TRIO domain of CRALBP binds to 11-*cis*-reti-nal, the critical component of the light-detecting rhodopsin in photoreceptor cells (He et al., 2009). The substrate-binding properties of MOSPD2 CRAL-TRIO are yet to be addressed.

Another interesting difference among VAP proteins is the variation in the linker lengths between their transmembrane regions and MSP domains (Figure 1b). VAPA and VAPB both contain coiled coil regions in their linkers with the longest calculated length of 26-27nm. MOSPD1, MOSPD2 and MOSPD3 have no predicted coiled coil regions and their calculated linker spans are shorter: 5, 18 and 12 nm, respectively. This suggests that individual VAP proteins can form MCS with varying distances between organelles, depending on how far the (phospho-)FFAT/FFNT motif of the interaction partner on the other membrane reaches. Also considering their motif selectivity and grouping, it is plausible to think that MOSPD1-MOSPD3 complexes form narrower MCS with respect to those formed by VAPA-VAPB-MOSPD2 complexes.

## Roles of VAP Proteins in Intercellular Signalling

It is predictable that by virtue of making numerous protein interactions, VAP proteins are involved in various biological processes besides forming MCS. These include ER-to-Golgi trafficking, unfolded protein response (UPR) and intercellular signalling (Kanekura et al., 2006; Prosser et al., 2008; Tsuda et al., 2008). The MSP domain of VAPB (and its homologs in Drosophila and nematodes) is secreted for intercellular signalling and VAPB fragments have been detected in blood serum (Tsuda et al., 2008). A survey of serum proteins also identified VAPA and VAPB in blood serum (Omenn, 2005; Tsuda et al., 2008). Interestingly, the P56S point mutant of VAPB that causes familial amyotrophic lateral sclerosis (ALS) cannot be secreted (Nishimura et al., 2004; Tsuda et al., 2008). Secreted VAPB can compete with ephrin proteins for the receptor tyrosine kinase EPHA4 (Tsuda et al., 2008). In the adult nervous system, ephrins are implicated in synapse formation and the regulation of long-term synaptic plasticity and memory (Klein, 2009; Van Hoecke et al., 2012). Genetic and pharmacological inhibition of EPHA4 increases survival in mouse and rat models of ALS (Van Hoecke et al., 2012). In human ALS patients, EPHA4 expression inversely correlates with disease onset and survival. Moreover, loss-of-function mutations in EPHA4 are associated with long survival in these patients. Based on these observations, it is possible that the pathological consequences of the ALS-causing P56S mutation arise not from its effect on intracellular VAPB function but from a dysfunction that involves intercellular ephrin signalling.

MOSPD2 has also been reported to be a cell surface receptor, with its N-terminus exposed to the extracellular space, involved in monocyte and neutrophil migrations (Mendel et al., 2017; Jiang et al., 2020). In the meantime, it remains unclear how VAPB and MOSPD2, two type-II membrane proteins with no signal peptide, are secreted or exposed to the extracellular space. A recent study demonstrated that proteins lacking a signal peptide can be sorted into secretory vesicles at the ER-Golgi intermediate compartment, ERGIC (Zhang et al., 2020). Possibly, VAPB and MOSPD2 are translocated to the extracellular side of cellular membranes using this or another unconventional mechanism. The frequency and efficiency of these unconventional secretion/translocation events and their role in health and disease are as yet to be addressed.

## VAP Proteins Involved in Genetic Diseases

Two point mutations in VAPB, T46I and P56S, have been identified as the leading cause of a rare form of familial ALS (Nishimura et al., 2004; Chen et al., 2010). Both mutations cause the hyper-ubiquitination of VAPB and promote the formation of large insoluble VAPB aggregates (Nishimura et al., 2004; Kanekura et al., 2006; Chen et al., 2010). While the VAPB P56S mutation does not affect the FFAT binding, overexpression of a FFAT motif peptide can rescue the aggregation phenotype of this mutant (Prosser et al., 2008). It is predicted that the P56S mutation causes insolubility by removing a kink between two short stretches of beta barrel strands (Nishimura et al.,2004). Interestingly, a corresponding P56S mutation in VAPA does not cause aggregation, suggesting a unique role of VAPB over VAPA in neuronal function (Prosser et al., 2008). Highlighting this notion, three additional VAPB mutations have been linked to ALS (van Blitterswijk et al., 2012; Kabashi et al., 2013). Additionally, VAPB levels are diminished in spinal motor neurons of ALS patients and lifelong neuronal overexpression of VAPB in ALS mouse models delayed loss of spinal motor neurons and extended lifespan (Teuling et al., 2007; Kim et al., 2016). It is yet to be established whether this is due to a function specific for VAPB or a process induced by VAPB mutations.

The newly identified VAP proteins MOSPD1 and MOSPD3 are also linked to diseases. A chromosomal duplication of the X-linked MOSPD1 locus is associated with double outlet right heart ventricle (Hirota et al., 2017). Similarly, MOSPD3 may play a role in right ventricle development (Pall et al., 2004). How these proteins are involved in heart development, is yet unclear.

## Intracellular Pathogens Hijack VAP-Mediated Contact Sites

**Figure 3:**
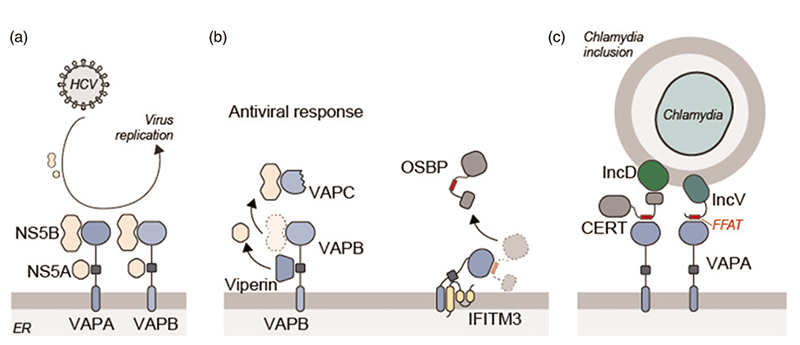
Intracellular pathogens take advantage of MCS formed by VAP proteins. (a) NS5A and NS5B proteins of HCV interact with VAPA and VAPB to locate viral replication machinery to the ER site. (b) Human anti-viral proteins Viperin and IFITM3 interact with VAPA/ VAPB to block their interaction with viral proteins or recruitment of host proteins to the replication site. (c) Bacterial proteins IncD and IncV recruit VAPA to form MCS between the ER and Chlamydia-containing inclusions.

As MCS form intracellular synapses where exchange of information and metabolites between intracellular compartments occur, a growing list of intracellular pathogens highjack these intracellular hubs. Rhinovirus relies on a PI-4-phosphate/cholesterol counter flow at the ER-Golgi interface to form replication compartments at these sites (Roulin et al., 2014). The norovirus proteins NS1 -which contains a FFAT motif-, NS2, and NS4 interact with VAPA (Figure 1d) (McCune et al., 2017). Furthermore, VAPA and VAPB recruit the hepatitis C virus (HCV) replication machinery to the ER (Figure 3a) (Shi et al., 2003; Gao et al., 2004). The HCV protein NS5A interacts with the coiled-coil regions of VAPA and VAPB; and the RNA polymerase NS5B interacts with the MSP domains of VAPA and VAPB (Tu et al., 1999; Gao et al., 2004; Hamamoto et al., 2005). The C-terminal flexible part of NS5B can associate with their MSP domains, while no FFAT motif was predicted in this region (Gupta and Song, 2016). Also, VAPC, a 99-residues long splice variant of VAPB that does not interact with FFAT motifs, binds to NS5B. This interaction impairs the contact between NS5B and VAPA/VAPB, leading to reduced HCV replication and virus propagation (Kukihara et al., 2009; Wen et al., 2011).

While VAP proteins are used by various viruses, they also contribute to anti-viral responses. The expression of the interferon-stimulated gene Viperin is upregulated in influenza, human immunodeficiency virus (HIV), dengue and HCV infections (Fitzgerald, 2011). Viperin inhibits HCV replication by interacting with the coiled coil region of VAPA, therefore interfering with the VAPA-NS5A interaction (Figure 3b) (Wang et al.,2012). Another interferon-stimulated gene, IFITM3 interacts with the coiled coil and transmembrane regions of VAPA to prevent its association with OSBP (Figure 3b). This leads to an accumulation of cholesterol in late endosomes and disrupts the fusion of viral particles with the late endosomal limiting membrane and thus entry into the cytosol (Amini-Bavil-Olyaee et al.,2013).

Using VAP proteins in the infectious cycle is not restricted to viruses. The obligate parasite *Chlamydia tramochatis* enters the cell by endocytosis. Then, Chlamydia-containing vesicles merge with late endosomes to create structures called inclusions. Replication of Chlamydia involves MCS between Chlamydia inclusions and the ER while various MCS proteins including VAPA are recruited to ER-inclusion contact sites (Elwell et al., 2011). The Chlamydia protein *IncD* interacts with the CERT PH domain to bring CERT and VAPA to these contact sites (Figure 3c) (Derré et al., 2011). Another Chlamydia protein *IncV* contains two FFAT motifs that allow interactions with VAPA/VAPB to bring the ER in close proximity with the Chlamydia inclusions (Figure 3c) (Stanhope et al., 2017). These interactions are critical in Chlamydia infection as VAP depletion impairs bacterial development (Derré et al., 2011). Overall, these findings summarize the critical role of VAP proteins play in viral and bacterial infections.

## Concluding Remarks and Perspectives

The interest in the study of MCS has multiplied over the years. In this review, we have summarized the latest developments regarding the VAP protein family, including the recently characterized VAP proteins and the newly identified motifs they interact with. VAP tethers operate in two segregated ER complexes: VAPA-VAPB-MOSPD2 and MOSPD1-MOSPD3 that bind to (phospho-)FFAT and FFNT motifs, respectively.

As the research in recent years has broadened the understanding of VAP-mediated MCS formation, many aspects of VAP proteins (related to MCS or not) still remain unknown. One question is why there are this many VAP proteins. A possible explanation is that more complex cellular life required intricate arrangement of its numerous contact sites, hence new VAP proteins and motifs have emerged throughout evolution. Characterization of new motifs in the form of FFNT and phospho-FFAT also raised the question whether other FFAT-related motifs are present. In addition, identification of the kinases/phosphatases that phos-phorylate/dephosphorylate phospho-FFAT -by which control the formation and duration of MCS between organelles-will work out the dynamics of MCS. It is also unclear whether other post-translational modifications and their related biology participate in motifs recognized by VAP proteins. Furthermore, as VAP proteins are involved in genetic and infectious diseases, a better understanding of VAP proteins may provide valuable insight in finding ways to control such diseases.

Another interesting aspect is the CRAL-TRIO domain of MOSPD2. It is unclear whether this domain contains lipid binding or lipid transfer property. As VAP proteins are appreciated for their ability to recruit lipid-binding and lipid transfer proteins to the ER, it remains puzzling why a VAP protein contains a domain of these potential properties (Hanada et al., 2003; Loewen et al., 2003; Rocha et al., 2009; Mikitova and Levine, 2012; Alpy et al., 2013; Mesmin et al., 2013; Weber-Boyvat et al., 2015; Di Mattia et al., 2018; Kumar et al., 2018).

As the cell can be considered as a society of interacting organelles orchestrated by the ER, the study of VAP proteins in motif binding, MCS formation, extracellular secretion, genetic and infectious diseases guarantees exciting research for years to come.
